# Prevalence and associated factors for poor mental health among young migrants in Sweden: a cross-sectional study

**DOI:** 10.1080/16549716.2023.2294592

**Published:** 2024-01-05

**Authors:** Sara Causevic, Anna Mia Ekström, Nicola Orsini, Anna Kagesten, Susanne Strömdahl, Mariano Salazar

**Affiliations:** a Department of Public Health Sciences, Stockholm University, Stockholm, Sweden; b Global and Sexual Health (GloSH) Research Group, Department of Global Public Health, Karolinska Institutet, Widerströmska huset, Stockholm, Sweden; c Department of Infectious Diseases, South Central Hospital, Stockholm, Sweden; d Department of Medical Sciences, Infectious Medicine, Uppsala University, Uppsala, Sweden; e Swedish Public Health Agency, Stockholm, Sweden

**Keywords:** Mental health, sexual health, refugees, adolescent, risk behaviour

## Abstract

**Background:**

Young migrants face multiple challenges that can affect their mental, sexual and reproductive health.

**Objective:**

To assess the prevalence of self-reported poor mental health and its associated demographic, post-migration and sexual risk behaviour factors among young migrants (aged 15–25) in Sweden.

**Methods:**

Data were drawn from a cross-sectional survey conducted with migrants aged 15–65 years old in Sweden between December 2018 and November 2019 (*n* = 6449). Among these, 990 participants aged 15–25 were eligible for the study. Mental health was measured using the Refugee Health Screener-13. Missing data indicator analysis and multivariable logistic regression models were conducted to estimate the association between mental health, sexual risk behaviour, demographic and migration-related variables.

**Results:**

Of the 990 participants, 59% reported poor mental health. Participants reporting poor mental health were more likely to be female (AOR:1.63, 95% CI:1.18–2.25), to have lived in Sweden more than three years (AOR:2.16, 95% CI:1.17–3.97), to engage in any sexual risk behaviour (AOR:1.99, 95% CI:1.25–3.17), and to live alone (AOR:1.95, 95% CI:1.25–3.03) or with friends they already knew (AOR:1.60, 95% CI:1.37–4.91). People arriving from the Americas (AOR:0.54, 95% CI:0.33–0.88), Asia (AOR:0.44, 95% CI:0.22–0.86), Europe (AOR:0.30, 95% CI:0.14–0.61) and Africa (AOR 0.37, 95% CI: 0.23–0.60) had lower odds of poor mental health than those arriving from Syria.

**Conclusion:**

The prevalence of poor mental health among young migrants in Sweden was high, with specific subgroups (women, asylum seekers, people arriving from Syria, and those residing longer in Sweden) being particularly vulnerable. Our results indicate the interconnectedness between poor mental health and sexual risk behaviour in this population. Thus, policies targeting young migrants should ensure that healthcare services screen for both poor sexual and mental health at the same time.

## Introduction

The World Health Organization (WHO) defines *mental health* as the state of well-being in which individuals can achieve their abilities and cope with everyday stress while working and living a productive life within their community [1]. Recent data found that mental disorders were among the top ten leading causes of the global burden of disease, with no evidence of the burden decreasing [2]. For example, the percentage of the population affected by major depressive or anxiety disorders ranged from 9.7% to 36% during the COVID-19 pandemic [3]. In addition, research has indicated a higher prevalence of mental health disorders such as post-traumatic stress and depression among certain migrant populations, such as refugees and asylum seekers [4].

Mental health is determined by a combination of individual characteristics (such as genetics and educational level), community conditions (including exposure to violence and poverty, etc.), and broader structural factors (climate change, limited access to health services, etc.) [5]. Among these, migration and forced displacement due to conflicts and political oppression have been recognised as the key structural factors for increasing the risk of poor mental health [5,6].

The global number of migrants in 2021 totalled 281 million, of which 11% were young migrants (≤25 years) [7]. Migrants comprise a heterogeneous group ranging from voluntary migrants to displaced people leaving their countries due to war, poverty, or political instability [8].

Migrants often face unique challenges predisposed by their migration experience, and these challenges are often shaped by contextual factors before, during, and after a person leaves their home [9–12]. For example, before migration, people can experience conflict-related trauma or feel a profound uncertainty about the safety of the journey. These experiences can lead to post-traumatic stress disorder (PTSD) and anxiety [10,11,13,14]. During migration, migrants, particularly vulnerable populations like women, children, young people, and LGBTQ+ individuals, can risk exploitation, discrimination, physical and sexual violence, and human trafficking [15–19]. After settling in the host country, factors such as cultural differences, language barriers, discrimination, uncertain legal status (such as lack of work or residence permit), lack of social networks, and limited access to health care have been shown to impair migrants´ mental health [9–11,14,20–24]. In particular, time living in the host country has been identified as a contributing factor to the increasing substance and alcohol abuse incidence among migrants living in Sweden [25]. Poor living conditions (e.g. living in refugee housing or being separated from family) were also associated with higher levels of psychological distress in a study conducted among refugees in Germany [26].

Poor sexual health, including sexual risk behaviours, is also associated with mental health and migration [15,16]. The interrelationship between sexual risk behaviour and mental health is complex, as one can catalyse the other [27,28]. For example, sexual risk behaviours in the form of unprotected sex with strangers can generate anxiety due to fears of contracting a sexually transmitted infection (STI) [29]. On the other hand, mental disorders and the medication used to treat them can increase sexual risk behaviours or impair sexual desire and people´s abilities to enjoy pleasurable sexual relations [28].

The challenges faced by many migrants in their host countries could influence their sexual health, including increased sexual risk behaviour. For example, engaging in sexual risk behaviours has been reported as a coping strategy to deal with feelings of loneliness and financial challenges or as a pathway to feel more integrated into a new society with more tolerant norms towards sex and sexuality [15,21,30–36]. Consequently, recognising and understanding the mental health challenges among young migrant populations is essential for improving their sexual and reproductive health (SRH) and vice versa [10,14,37].

Sweden is a multicultural society where approximately 19.7% of Swedish residents are foreign-born [38]. Previous studies have shown that first- and second-generation young migrants have an increased risk for poor mental health compared to their Swedish-born peers [22,24,39]. For instance, young refugees diagnosed with poor mental health have a higher risk of PTSD and reported lower medication uptake than their Swedish-born counterparts [24,40]. Although previous studies in Sweden have provided key data on the demographic risk factors (e.g. age, age at migration, country of origin, education, etc.) for poor mental health among this vulnerable population, few studies have assessed if sexual risk behaviours, post-migration conditions (living arrangements, years living in Sweden, reason for migration and residency status) increase the odds of poor mental health among young migrants in Sweden [22,24,40]. Thus, our study aimed to fill this research gap by mapping the prevalence of self-reported poor mental health and its associated demographic, post-migration, and sexual risk behaviours factors among young migrants (aged 15–25) in Sweden. Our findings will help inform policies to design comprehensive health services for young migrants, including mental and sexual health screening and treatment in Sweden and elsewhere.

## Methods

### Study population

The data used for this paper comes from a larger project that aimed to map the sexual, reproductive, and mental health of migrants aged 15–65 years living in Stockholm, Sweden [31,41]. A migrant was defined as ‘any person changing their country of residence who has moved from their usual place of residence, either in-country or across the border, for various reasons’ [8].

#### Sample and data collection

Data for the project above were collected between December 2018 and November 2019 using an anonymous, self-administered cross-sectional survey. The target population was migrants attending schools offering Swedish language training for foreigners and upper-middle secondary schools in Stockholm. The data was collected in collaboration with two non-governmental organisations (NGOs), The World Values Survey (WVS) and Invandraindex [42,43]. Both NGOs collect health, demographic, and migration-related data from thousands of migrants in Sweden. In total, 6449 people aged 15–65 answered the survey. We excluded 180 people who did not consent to participate in the survey. For our paper, we selected only those aged 15–25 years, thus excluding 4706 people and 573 who did not complete all the questions for Refugee Health Screener-13 (RHS-13), which is the main tool for the outcome of this paper (see full definition and description below). Thus, the final sample for analysis was 990 participants (see Supplementary file, Figure 1).

**Figure 1.**
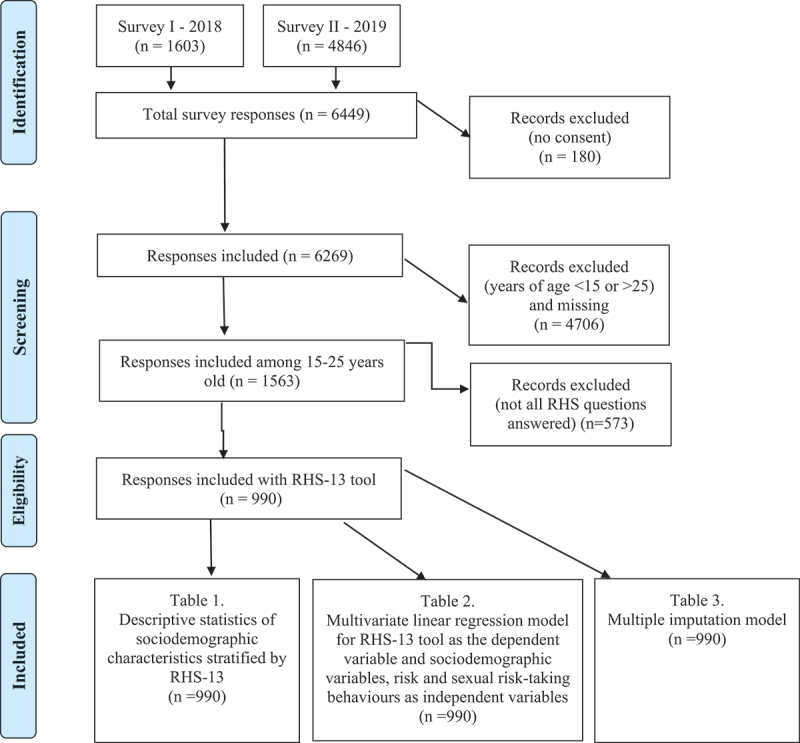
Flowchart of the number of participants, responses, and tables.

#### Data collection

The survey was available in Arabic, English, Farsi, Tigrinya, Spanish, Swedish, and Somali. It was tested in 2018 for language correctness, translated, and back-translated for accuracy. The survey was administered in the teacher’s classroom, where the participants answered it using a personal or school-provided digital device. A research assistant was present to clarify any questions. The participants were given approximately 60 minutes to complete the survey. Additional details on participant recruitment and the study sample are described elsewhere [31,41].

### Variables

#### Dependent variable

The main outcome variable, self-reported mental health, was measured using the RHS-13 [44]. The RHS-13 is a self-rating screening instrument used to identify people with high risk for mental health problems. It has been used and validated in Sweden and elsewhere [45,46]. The RHS-13 has good internal validity (Cronbach’s α = 0.95), sensitivity, and specificity when compared to other specific mental health diagnostic tools (PTSD: sensitivity 0.81/specificity 0.87, anxiety: 0.94/0.86, and depression: 0.95/0.89) [44]. The RHS-13 consists of 13 questions describing poor mental health signs and symptoms rated using a Likert scale ranging from 0 = not at all to 4 = extreme. The overall scale ranges from 0 to a maximum score of 52. As suggested by the literature, a cut-off of 11 points or above on the scale was used to define poor mental health [44].

#### Independent variables

##### Sexual risk behaviour

Past-year sexual risk behaviour (yes, no, haven´t had sex) was assessed by combining the positive responses to any of the following three questions: 1) had sex without a condom; 2) had sex in exchange for gifts/money; or 3) had sex with a casual partner under the influence of drugs. We combined these variables to facilitate analysis since it has been reported that sexual risk behaviours among young people usually cluster together [47].

##### Demographic variables

We measured demographic variables previously shown to influence mental health [24,45,48]: Sex (*male, female);* Age (*years);* Education measured as years in school (*<3 years, 4–6 years, 7–9 years, 10 years or longer);* Religion (*Christianity, Islam, no religion/other);* and Country of birth, grouped into seven categories based on the main settings that participants came from *1) Asia, 2) Americas, 3) Europe, 4) Africa, 5) the Middle East, North Africa (MENA, including Algeria, Bahrain, Egypt, Iran, Iraq, Israel, Jordan, Kuwait, Lebanon, Libya, Morocco, Oman, Qatar, Saudi Arabia, Palestine, Tunisia, United Arab Emirates, Yemen), 6) Syria, and 7) Afghanistan*. Syria and Afghanistan were analysed separately (Syria was excluded from MENA region countries) due to the more significant number of survey participants (28% and 16%, respectively) that came as refugees and asylum seekers to Sweden in 2016 (when the study data was collected) [49,50]. Under Africa, we predominantly mean countries of sub-Saharan Africa.

##### Post-migration-related variables

We measured four variables: Main reason for coming to Sweden (*asylum seeker/refugee*, *work/study*, *family reunion, other*), Having a Swedish residence permit (*no*, *yes*, *already an EU/EEA resident*), Number of years living in Sweden (<1 year, 1, 2, 3, 4, or ≥5 years), and Current living arrangements (*alone*, *married or cohabiting*, *other family*, with *friends they already knew*, or *refugee home*).

### Analysis

The descriptive analysis results are reported as percentages, means, and standard deviation (SD). Pearson chi-square and t-tests were used to compare differences between groups.

Multivariable logistic regression was used to estimate adjusted odds ratios (AOR) and 95% confidence intervals (CIs) for the association between poor mental health (dichotomised RHS-13 score) and the independent variables. Independent variables were included in the model if they have been shown to be associated with poor mental health among this population [10,14,22,23,39].

The independent variables had different patterns of missingness ranging from 12% for demographic variables up to 56% for the sexual risk behaviour variable. Thus, we used a missing data indicator analysis to use the entire data set containing the complete RHS-13 score by adding the option ‘missing’ to all covariables with missing data. As a sensitivity analysis, we conducted multiple imputations of all categorical variables assumed to be missing at random using chained equations (MICE) and estimates combined with Rubin’s rule [51,52]. Data were analysed using Stata version 17 (StataCorp, College Station, Texas).

## Ethics and consent

The study was approved by the Regional Ethical Review Board in Stockholm (2017/2030–31 and 2018/1002–32) and follows the ethical principles of the Declaration of Helsinki 1964. The participants were given verbal and written information about the study and informed that participation was voluntary and anonymous and that they could quit at any time. According to Swedish law, children aged 15 years or above could participate in the study without parental/guardian consent (The Act on Ethical Review, §18-Lag (2003:460)).

## Results

### Sample characteristics

Among the study participants, 54% were male. The mean age was 19.6 years (SD = 2.7) (Table 1). A third (29%) had resided in Sweden for four years and a third came from Syria (27%), followed by Africa (18%). Most came as asylum seekers/refugees (53%). Of those reporting having had sex (*n* = 435), 35% had any sexual risk behaviours in the last year (Table 1).

**Table 1.** Descriptive statistics of the study cohort (15–25 years old) and bivariate analysis of independent variables by RHS-13 (n = 990).

	All participants	Mental health (RHS-13 < 11)	Poor mental health (RHS-13 ≥ 11)
Characteristics	n (%)	n (%)	n (%)
**Sex**			
Male	531 (53.6)	214 (53.3)	317 (54.6)
Female	445 (45.0)	190 (46.4)	255 (43.9)
Missing	14 (1.4)	5 (1.2)	9 (1.5)
*Total*	*990 (100)*	*409 (100)*	*581 (100)*
**Age. Mean (SD)**	19.6 (2.7)	19.6 (2.9)	19.6 (2.6)
Education (Years)*			
<3 years	194 (19.6)	54 (13.2)	140 (24.1)
4–6	212 (214)	83 (20.3)	129 (22.2)
7–9	124 (12.5)	56 (13.7)	68 (11.7)
10 years or longer	439 (44.3)	207 (50.6)	232 (39.9)
Missing	21 (2.1)	9 (2.2)	12 (2.1)
*Total*	*990 (100)*	*409 (100)*	*581 (100)*
Number of years living in Sweden (Years)*			
<1 year	114 (11.5)	68 (16.6)	46 (7.9)
1 year	209 (21.1)	104 (25.4)	105 (18.1)
2 years	207 (20.9)	105 (25.7)	102 (17.7)
3 years	111 (11.2)	43 (10.5)	68 (11.7)
4 years	284 (28.7)	65 (15.8)	219 (37.7)
5 years	59 (5.9)	24 (5.9)	35 (6.0)
Missing	6 (0.6)	0 (0)	6 (1.0)
*Total*	*990 (100)*	*409 (100)*	*581 (100)*
Religion*			
Not religious/Other	165 (16.7)	68 (16.6)	97 (16.7)
Christianity	183 (21.1)	93 (22.7)	90 (15.5)
Islam	575 (58.1)	216 (52.8)	359 (61.8)
Missing	67 (6.8)	3 (7.8)	35 (6.0)
*Total*	*990 (100)*	*409 (100)*	*581 (100)*
Country of birth*			
Asia	59 (6.0)	33 (8.1)	26 (4.5)
Americas	124 (13.0)	59 (14.4)	65 (11.2)
Europe	76 (8.0)	47 (11.5)	29 (5.0)
Africa	171 (18.2)	96 (23.5)	75 (12.9)
MENA	127 (13.4)	40 (9.8)	87 (15.0)
Syria	253 (26.6)	90 (22.0)	163 (28.1)
Afghanistan	140 (14.7)	27 (6.6)	113 (19.5)
Missing	40 (4.0)	17 (4.2)	23 (4.0)
*Total*	*990 (100)*	*409 (100)*	*581 (100)*
**Having a residence permit in Sweden**			
No	63 (6.4)	19 (4.6)	44 (7.6)
Yes	649 (65.6)	271 (66.3)	378 (65.1)
I am an EU/EEA/Swedish citizen	30 (3.0)	17 (4.2)	13 (2.2)
Missing	248 (25.0)	102 (25.0)	146 (25.1)
*Total*	*990 (100)*	*409 (100)*	*581 (100)*
The main reason to come to Sweden*			
I came as an asylum seeker/refugee	523 (52.8)	174 (42.5)	349 (60.1)
I came to work/study	99 (9.9)	46 (11.2)	52 (8.9)
I came to live with family	190 (19.2)	97 (23.7)	93 (16.0)
Other	71 (7.2)	36 (8.8)	35 (6.0)
Missing	108 (11.0)	56 (13.7)	52 (8.9)
*Total*	*990 (100)*	*409 (100)*	*581 (100)*
Current living arrangements*			
Alone	186 (18.8)	58 (14.2)	128 (22.0)
Married or cohabiting	136 (13.7)	60 (14.7)	76 (13.1)
With other family	549 (55.4)	258 (63.1)	291 (50.1)
With friends they already knew	83 (8.4)	18 (4.4)	65 (11.2)
In a refugee home	21 (2.1)	6 (1.5)	15 (2.6)
Missing	15 (1.5)	9 (2.2)	6 (1.0)
*Total*	*990 (100)*	*409 (100)*	*581 (100)*
Sexual risk behaviour*			
Not taking sexual risks	283 (28.6)	121 (29.6)	162 (27.9)
Taking sexual risks	152 (15.3)	48 (11.7)	104 (17.9)
Missing	555 (56.1)	240 (58.7)	315 (54.2)
*Total*	*990 (100)*	*409 (100)*	*581 (100)*

*p < 0.05, Comparison between those reporting values above and equal to 11, chi2 test.

n = number of observations. M = Mean. SD = Standard Deviation. RHS-13: Refugee Health Screener-13.

MENA countries (Algeria, Bahrain, Egypt, Iran, Iraq, Israel, Jordan, Kuwait, Lebanon, Libya, Morocco, Oman, Qatar, Saudi Arabia, Palestine, Syria (excluded), Tunisia, United Arab Emirates, Yemen).

### Bivariate analysis

The prevalence of poor mental health was 59% (55% in male and 44% in female participants). Poor mental health (RHS-13 score ≥ 11) was significantly associated (p < 0.05) with education, the number of years of living in Sweden, religion, country born and raised, reasons to come to Sweden, current living arrangements, and sexual risk behaviour. There was no bivariate association with sex and residence permit in Sweden (Table 1).

### Multivariable logistic regression and multiple imputation analysis

Results from the multivariable logistic regression analysis are shown in Table 2, Model 1. Factors associated with increased odds for poor mental health were: being female (OR 1.63, 95% CI:1.18–2.25), living in Sweden for more than three years (OR: 2.16, 95% CI: 1.17–3.97), engaging in any sexual risk behaviours (OR:1.99, 95% CI: 1.25–3.17) and living alone (OR:1.95, 95% CI: 1.25–3.03) or with friends they already knew (OR: 2.60, 95% CI: 1.37–4.91) compared to living with other family members. Coming from Asia, the Americas, Africa, and Europe decreased the odds of poor mental health compared to coming from Syria.

**Table 2.** Association between demographic, migration-related, and sexual risk behaviour variables with poor mental health (RHS-13 ≥ 11); adjusted odds ratios (AOR) and 95% confidence intervals (CI) shown (n = 990).

Characteristics	Model 1. Multivariable logistic regression (*n* = 990)	Model 2. Multiple imputation (*n* = 990)
**Sex**		
Male	1.00	1.00
Female	1.63 (1.18–2.25)*	1.70 (1.22–2.37)*
Missing	1.87 (0.58–6.08)	
**Age (years)**. Mean (SD)	0.99 (0.93–1.06)	0.99 (0.93–1.06)
**Education (years)**		
3 or less	1.00	1.00
4–6	0.69 (0.43–1.10)	0.75 (0.45–1.5)
7–9	0.62 (0.35–1.09)	0.62 (0.35–1.09)
10 or longer	0.68 (0.43–1.07)	0.66 (0.41–1.06)
Missing	0.58 (0.21–1.66)	
**Years living in Sweden**		
Less than one year	1.00	1.00
1 year	1.45 (0.87–2.42)	1.40 (0.83–2.35)
2 years	1.43 (0.84–2.42)	1.32 (0.77–2.27)
3 years	2.16 (1.17–3.97)*	1.83 (0.97–3.45)
4 years	2.87 (1.63–5.03)*	2.62 (1.45–4.74)*
5 years or longer	2.12 (1.02–4.36)*	1.70 (0.79–3.64)
Missing	(empty)	
**Religion**		
Not religious/other	1.00	1.00
Christianity	0.88 (0.52–1.47)	0.84 (0.48–1.48)
Islam	0.97 (0.63–1.49)	0.93 (0.59–1.47)
Missing	0.59 (0.29–1.13)	
**Country of birth**		
Syria	1.00	1.00
Asia	0.44 (0.22–0.86)*	0.41 (0.20–0.82)*
Americas	0.54 (0.33–0.88)*	0.50 (0.29–0.84)*
Europe	0.30 (0.14–0.61)*	0.35 (0.15–0.77)*
Africa	0.37 (0.23–0.60)*	0.35 (0.21–0.57)*
MENA	0.84 (0.58–1.39)	0.74 (0.45–1.23)
Afghanistan	1.04 (0.18–1.87)	0.95 (0.52–1.73)
Missing	0.41 (0.18–0.90)	
**Residence permit**		
EU/EEA citizen	1.00	1.00
No	2.01 (0.67–6.03)	2.54 (0.87–7.42)
Yes	0.87 (0.33–2.30)	1.12 (0.41–3.04)
Missing	0.93 (0.35–2.47)	
**Main reason to come to Sweden**		
Asylum seeker/refugee)	1.00	1.00
To work/study	1.07 (0.62–1.83)	1.00 (0.57–1.75)
To live with family	0.68 (0.33–1.05)	0.59 (0.35–1.01)
Other	0.76 (0.35–1.37)	0.61 (0.31–1.22)
Missing	0.73 (0.42–1.28)	
**Current living arrangements**		
With other family members	1.00	1.00
Alone	1.95 (1.25–3.03)*	1.75 (1.10–2.77)*
Married or cohabiting	1.22 (0.75–1.96)	1.05 (0.64–1.74)
With friends they already knew	2.60 (1.37–4.91)*	2.58 (1.35–4.91)*
In a refugee home	1.54 (0.54–4.42)	1.31 (0.44–3.92)
Missing	0.52 (0.16–1.66)	
**Sexual Risk behaviour**		
Not taking sexual risks	1.00	1.00
Taking sexual risks	1.99 (1.25–3.17)*	2.30 (1.42–3.75)*
Missing	1.12 (0.81–1.56)	

n = number of observations. M = Mean. SD = Standard Deviation, *p < 0.05, RHS-13: Refugee Health Screener-13. *MENA countries (Algeria, Bahrain, Egypt, Iran, Iraq, Israel, Jordan, Kuwait, Lebanon, Libya, Morocco, Oman, Qatar, Saudi Arabia, Palestine, Syria (excluded), Tunisia, United Arab Emirates, Yemen).

The multiple imputation analysis (Table 2, Model 2) showed that all variables significant in Model 1 had the same direction of association (risk or protective factor) and similar magnitude (AOR) and confidence intervals in the imputed analysis.

## Discussion

Our main findings showed that six out of ten young migrants screened positive for poor mental health. Our multivariable analysis identified several factors associated with our main outcome. We observed increased odds for poor mental health in female rather than male participants. Living in Sweden for four years compared to living for less than one year and engaging in sexual risk behaviour in the last 12 months increased the odds of reporting poor mental health. Lastly, in those currently living alone or with friends they already knew, we observe higher odds of poor mental health than those living with family. Compared to those arriving from Syria, participants reporting to come from the Americas, Africa, Europe, or Asia had lower odds of poor mental health. In the following, we will discuss these findings in depth.

The high prevalence of poor mental health in our study sample aligns with previous studies in Sweden and elsewhere [22,23,33,53–55]. Our finding can be explained by the composition of our sample, where 53.8% were refugees or asylum seekers, and 28% reported Syria as their home country. This migrant subgroup is likely to have been exposed to conflict-related trauma, as reported in other studies globally [22,24,40,56–58]. During migration, refugees and asylum seekers often face exclusion, discrimination, financial challenges, and exposure to different forms of violence (physical, sexual, etc.), which can be detrimental to migrants´ mental health [22,33,59]. In Sweden, previous studies have found that refugees had a higher risk of schizophrenia and psychiatric hospitalisation than other migrant groups [39,60]. The discussion above can also explain why, when compared to Syria, arriving from other geographical regions was a protective factor against poor mental health in our study findings.

In our study, females had higher odds of poor mental health than males, in line with previous studies conducted in Europe and Sweden [58,61,62]. One possible explanation is the gendered exposure to violence that migrants (and people in general) face. For example, one study found that female refugees were more likely to report sexual violence and family violence (from a partner or other family member) than males [63]. Another possible explanation is that female migrants´ dependency on their partner for a visa or residency permit can be used by violent partners/family members to control and abuse them, as reported in an Australian study and elsewhere [64–66]. The gendered nature of our findings implies that programmes aiming to improve the mental health of migrants must make efforts to prioritise women in their interventions.

Our findings also showed that the risk for poor mental health increased with the number of years living in Sweden. This is in line with another study conducted in Sweden showing a decline in the mental health of migrant populations, especially those with culturally distinct backgrounds from Sweden [22]. A possible explanation for our results is that as migrants spend more time in Sweden, they are faced with new challenges associated with the acculturation process, higher exposure to discrimination, limited access to health services, delayed resolution of the migration status, family separation, and poor employment opportunities which will negatively impact their mental health [13,22,67]. As mentioned, these challenges can have an impact on their sexual risk behaviours, causing a vicious cycle of poor health. Thus, policies aiming to improve the mental health of migrants must include long-term follow-ups and provide appropriate access to mental health services.

The living arrangements of migrants in our data were also associated with their poor mental health. Those living alone or with friends had worse mental health than those living with family members, which aligns with the findings reported in other studies [9,13,33,68]. A possible explanation for this finding is loneliness due to family separation and lack of instrumental or emotional family support, as shown in other studies [10,34,53,69].

Our analysis also showed that those engaging in sexual risk behaviours had higher odds of poor mental health than those who did not. Our findings align with studies conducted in Sweden and elsewhere [16,21,70,71]. One possible explanation is that in the acculturation process, migrants could adopt a more open stance to casual sex and other sexual risk behaviours to integrate better into the new society [21]. However, these risky behaviours can be detrimental to young people´s mental health as they might worry about the negative social and health consequences of their actions, as shown in a study among youth in Sweden [29]. The cross-sectional nature of our data does not allow us to establish a temporal or causal association; however, the association between poor mental health and sexual risk behaviours must be addressed in intervention aiming to tackle poor mental health among migrants. This means mental health services should consider screening for sexual risk behaviours and vice versa.

## Limitations

The generalisation of our results is limited since our sample did not include migrants with lower literacy levels, undocumented, or those not attending Swedish language schools. In addition, the prevalence of poor mental health in our study might be underestimated if those excluded from the analysis due to incomplete data on the outcome variable had worse mental health than those completing the data. The cross-sectional nature of our data did not allow us to establish a causal association between the outcome and independent variables. Questions about behaviours during the last 12 months may produce recall bias.

## Conclusions

Our findings highlight the prevalence of poor mental health among young migrants in Sweden, with specific subgroups, such as young women, asylum seekers, and those residing longer in Sweden, being particularly vulnerable. Furthermore, our results indicate the interconnectedness with sexual risk behaviour among young migrants. These findings have important implications for the Swedish healthcare system.

First, policies and programmes must ensure young migrants’ right to health, including the availability and accessibility of mental health services. Second, mental care services should consider policies to detect and address sexual risk behaviours in this population. Third, policies addressing the needs of vulnerable subgroups must be strengthened, considering the higher risk of poor mental health than others.

Our findings also call for longitudinal and qualitative research to better understand and address factors predisposing young migrants to poor mental and sexual health. Such research can inform the development of effective interventions and strategies.

## Supplementary Material

Flow diagram.doc

Author Bio.docx

## Paper context

Young migrants in Sweden can face challenges impacting their mental, and sexual and reproductive health. We assessed a sample of 990 migrants aged 15–25 for mental health prevalence, including associations with sexual risk behaviours and migration-related factors. Poor mental health (prevalence = 59%) was linked to female gender, longer stay in Sweden, sexual risk behaviour, risk propensity, and lower education levels. These findings call for accessible mental health services, prevention of risk behaviour, and targeted support.
