# Supplementary material for: Prevalence and associated factors for poor mental health among young migrants in Sweden: a cross-sectional study
Source: Glob Health Action. 2024 Jan 5;17(1):2294592. doi: 10.1080/16549716.2023.2294592 (PMC10773640; doi:10.1080/16549716.2023.2294592)

**Figure 1. Flowchart of the number of participants, responses, and tables**

**Identification**

Survey I - 2018
(n = 1603)

Survey II - 2019

(n = 4846)

Total survey responses (n = 6449)

Records excluded

(no consent)
(n = 180)

**Screening**

Responses included (n = 6269)

Records excluded

(years of age <15 or >25) and missing
(n = 4706)

Responses included among 15-25 years old (n = 1563)

Records excluded

(not all RHS questions answered) (n=573)



**Eligibility**

Responses included with RHS-13 tool

(n = 990)

**Included**

Table 2.

Multivariate linear regression model for RHS-13 tool as the dependent variable and sociodemographic variables, risk and sexual risk-taking behaviours as independent variables

(n =990)

Table 1.

Descriptive statistics of sociodemographic characteristics stratified by RHS-13
(n =990)

Table 3.

Multiple imputation model
(n =990)


Supplement: Flow diagram.doc [file ZGHA_A_2294592_SM3739.doc]
