# Supplementary material for: Prevalence and associated factors for poor mental health among young migrants in Sweden: a cross-sectional study
Source: Glob Health Action. 2024 Jan 5;17(1):2294592. doi: 10.1080/16549716.2023.2294592 (PMC10773640; doi:10.1080/16549716.2023.2294592)
Supplement: Author Bio.docx [file ZGHA_A_2294592_SM3738.docx]

**Author Bio**

**Sara Causevic** is a Postdoctoral fellow at the Department of Public Health Sciences at Stockholm University, Sweden. She is a health policy and systems researcher with a PhD in Medical Science from Karolinska Institutet, Sweden. In addition,she is a Commissioner with the Lancet Commission on Peaceful Societies through Health and gender equality. Her interest is in policy, governance, health systems and conflicts research.
